# Glycogen synthase kinase 3 has a limited role in cell cycle regulation of cyclin D1 levels

**DOI:** 10.1186/1471-2121-7-33

**Published:** 2006-08-30

**Authors:** Ke Yang, Yang Guo, William C Stacey, Jyoti Harwalkar, Jonathan Fretthold, Masahiro Hitomi, Dennis W Stacey

**Affiliations:** 1 From the Department of Molecular Genetics, The Lerner Research Institute, The Cleveland Clinic Foundation, 9500 Euclid Avenue, Cleveland OH, USA; 2 The Department of Neurology, Hospital of the University of Pennsylvania, Philadelphia, PA, USA

## Abstract

**Background:**

The expression level of cyclin D1 plays a vital role in the control of proliferation. This protein is reported to be degraded following phosphorylation by glycogen synthase kinase 3 (GSK3) on Thr-286. We recently showed that phosphorylation of Thr-286 is responsible for a decline in cyclin D1 levels during S phase, an event required for efficient DNA synthesis. These studies were undertaken to test the possibility that phosphorylation by GSK3 is responsible for the S phase specific decline in cyclin D1 levels, and that this event is regulated by the phosphatidylinositol 3-kinase (PI3K)/AKT signaling pathway which controls GSK3.

**Results:**

We found, however, that neither PI3K, AKT, GSK3, nor proliferative signaling activity in general is responsible for the S phase decline in cyclin D1 levels. In fact, the activity of these signaling kinases does not vary through the cell cycle of proliferating cells. Moreover, we found that GSK3 activity has little influence over cyclin D1 expression levels during any cell cycle phase. Inhibition of GSK3 activity by siRNA, LiCl, or other chemical inhibitors failed to influence cyclin D1 phosphorylation on Thr-286, even though LiCl efficiently blocked phosphorylation of β-catenin, a known substrate of GSK3. Likewise, the expression of a constitutively active GSK3 mutant protein failed to influence cyclin D1 phosphorylation or total protein expression level.

**Conclusion:**

Because we were unable to identify any proliferative signaling molecule or pathway which is regulated through the cell cycle, or which is able to influence cyclin D1 levels, we conclude that the suppression of cyclin D1 levels during S phase is regulated by cell cycle position rather than signaling activity. We propose that this mechanism guarantees the decline in cyclin D1 levels during each S phase; and that in so doing it reduces the likelihood that simple over expression of cyclin D1 can lead to uncontrolled cell growth.

## Background

Cyclin D1 plays a critical role in the regulation of proliferation by adjusting its expression levels to reflect the proliferative signaling environment of the cell, and then by regulating the cell cycle control machinery accordingly[1]. Cyclin D1 functions primarily to bind and activate the cyclin dependent kinase (CDK) 4/6, which then phosphorylates the retinoblastoma protein (Rb). Upon phosphorylation Rb releases the transcription factor E2F, which is then able to activate the transcription of genes required for G1/S phase transition[2-5]. The cyclin D1/CDK4/6 complex is also able to sequester p27^kip1 ^and other CDK inhibitory proteins, thereby neutralizing their inhibitory capacity for cyclin E/CDK2[6] whose activity is required for G1/S transition[7,8].

The regulation of cyclin D1 activity is primarily dependent upon its expression level. This level is controlled by the regulation of gene expression, mRNA stability and translation, and by protein stability. Cyclin D1 mRNA synthesis is regulated by mitogenic signaling pathways downstream of Ras activity. These include the Raf-1, MEK1/2 and ERKs pathways[9-11] ; along with the Ral and Rac GTPases [12,13]. Translational control of cyclin D1 is also under the control of growth factor signaling through activation of the eukaryotic initiation factor 4E, an effector of the phosphatidylinositol-3 kinase (PI3K)/AKT/mTOR signal pathway [14]. The stability of cyclin D1 protein also plays a major role in the regulation of its expression. Phosphorylation on Thr-286 has been reported to result in rapid proteasomal degradation of cyclin D1 [15]. It is also possible that this phosphorylation results in the export of cyclin D1 from the nucleus where it is functionally inactivated due to separation from its nuclear substrates [16]. In either case, the kinase responsible has been reported to be glycogen synthase kinase 3 (GSK3), which is an excellent in vitro kinase for cyclin D1 Thr-286 [17]. GSK3 is presumed to be constitutively active and therefore able to suppress cyclin D1 levels until phosphorylated. This phosphorylation can be carried out by AKT, which is in turn activated by PI3K [18,19], suggesting that the PI3K/AKT/GSK3 pathway controls cyclin D1 stability [15,17].

Not only are overall cyclin D1 levels critical in the growth properties of the cell, the levels of this protein are actively regulated through the cell cycle. We observed this fact using quantitative image analysis of antibody stained asynchronous cultures. Cyclin D1 expression was found to be high in G1 and G2 phase cells, but fell to low levels during S phase [20]. Subsequent studies have demonstrated that this expression pattern is vital to the regulation of ongoing cell cycle progression. The elevation of cyclin D1 during G2 phase depends upon proliferative signaling, and is required for the continuation of cell cycle progression [21,22]. Suppression of cyclin D1 during S phase is required for DNA synthesis, since high cyclin D1 levels are reported to bind PCNA and are able to block DNA synthesis [23,24]. The requirement that cyclin D1 levels fall during S phase is likely to restrict the chance of uncontrolled proliferation resulting simply from the elevated expression of cyclin D1 [21]. Critically, we have found that the specific suppression of cyclin D1 levels during S phase is dependent upon phosphorylation of Thr-286, since a mutation at this position blocked S phase cyclin D1 suppression [24]. These studies were undertaken to test the possibility, suggested by the above considerations, that the phosphorylation on Thr-286 responsible for the suppression of cyclin D1 during S phase is catalyzed by GSK3.

If GSK3 were responsible for the S phase suppression of cyclin D1, its activity would likely be lower during G1 and G2 phases than during S phase. One mechanism for achieving this cell cycle regulation of GSK3 activity would be for the activities of PI3K and AKT to be modulated through the cell cycle. There is a precedent for such cell cycle variations in proliferative signaling. For example, when oncogenic Ras is introduced into NIH3T3 cells, cyclin D1 levels are rapidly induced, but only during G2 phase [25]. Moreover, signaling downstream of Ras activity suppresses p27Kip1 (p27) levels throughout the cell cycle, but this is accomplished during each cell cycle phase through the activation of separate signaling molecules [26]. However, we find here that neither the activity of proliferative signaling kinases nor of GSK3 itself varies through the cell cycle. In fact, we find that GSK3 is not responsible for the suppression of cyclin D1 levels during S phase. Rather, we now postulate that cyclin D1 suppression during S phase is the result of cell cycle position rather than proliferative signaling.

## Results

### The cyclin D1 expression pattern is not altered by signaling inhibitors

**Figure 1:**
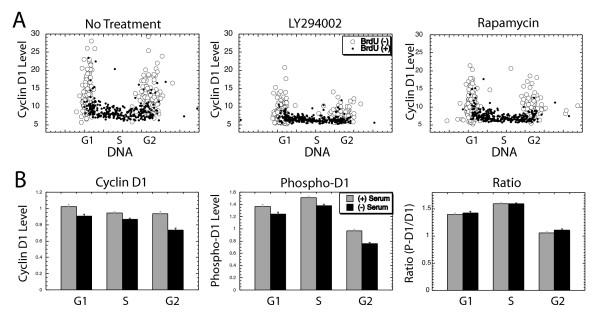
Cyclin D1 expression following inhibitor treatment: (A) MRC5 cells were left untreated (left) or were treated with 20 μM LY294002 (middle) to inhibit PI3K or 50 nM rapamycin (right) to inhibit mTOR. After 2 hrs cyclin D1, BrdU added as a final pulse, and DNA were stained and subjected to image analysis. Cyclin D1 levels for individual cells are plotted vs. DNA levels, with BrdU positive, S phase cells shown as small, solid circles; with cell cycle phases noted. (B) Serum was removed from NIH3T3 cultures for 4 hrs, after which cells were fixed and stained for total cyclin D1 and with an antibody able to recognize phospho-Thr-286 cyclin D1. The average cyclin D1 (left) or phospho-Thr-286 cyclin D1 (middle) for each cell cycle phase was determined and plotted (darker bars), together with the similar value determined from a parallel culture maintained in serum (lighter bars). Finally (right) the ratio of phospho-cyclin D1 to total cyclin D1 is presented for both the normal and serum deprived culture.

If the PI3K/AKT/GSK3 pathway stabilizes cyclin D1 levels specifically during G1 and G2 phases as suggested above, inhibitors of this pathway would produce a reduction in cyclin D1 expression during these cell cycle phases to the low levels seen during S phase. Thus, inhibition of these signaling pathways would be expected to result in low, uniform expression of cyclin D1 throughout the cell cycle. PI3K was inhibited by LY294002, while the kinase mTOR was inhibited by rapamycin in actively cycling human diploid fibroblast (MRC5) cultures. After 2 hrs treatment, including a terminal pulse with BrdU, the culture was fixed and stained with fluorescent antibodies against both cyclin D1 and BrdU, while DNA was stained with DAPI. Individual images of each fluorochrome were collected with a sensitive CCD camera, and subjected to image analysis to accurately quantitate the level of each fluorochrome in each cell (see [20]). The results were displayed by plotting the cyclin D1 level of each cell against its DNA level, with BrdU positive cells noted (Fig. 1A). Treatment with LY294002 dramatically reduced cyclin D1 levels, yet the overall cell cycle pattern of expression was maintained, with higher levels observed during G1 and G2 phases as in untreated cultures. Rapamycin had a similar but weaker effect on the cyclin D1 level (Fig. 1A). Neither inhibitor produced the uniform cyclin D1 expression through the cell cycle we had predicted.

**Figure 2:**
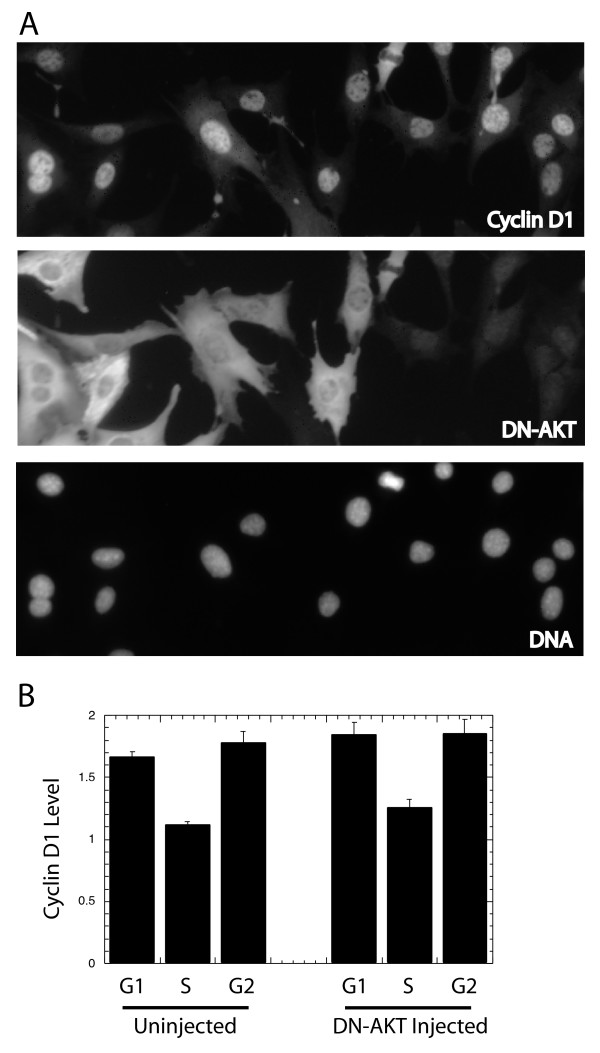
Dominant inhibitory AKT and cyclin D1 expression: (A) A plasmid expressing dominant inhibitory AKT was microinjected into NIH3T3 cells at the left of the area shown. The cells were incubated 8 hrs, fixed and stained with a fluorescent antibody stain against cyclin D1 (top), AKT (middle), or with DAPI to stain DNA (bottom). The same area of cells is shown in each frame. (B) NIH3T3 cells were injected with the inhibitory AKT plasmid 24 hrs prior to fixation and staining as above. Injected and uninjected cells were identified by staining for AKT, and cells were divided into cell cycle phase according to BrdU labeling and DNA content. The average cyclin D1 levels are shown for each group of cells.

We next inhibited AKT activity by microinjecting a plasmid expressing a dominant inhibitory mutant. This plasmid, pCDNA3-Akt-DN (K179 M, a kind gift of Dr. Nissim Hay) generates a kinase-dead AKT protein that can be detected within the cells by antibody staining. Staining confirmed the high level expression of the mutant protein at 4 and 24 hrs following plasmid injection. This protein, however, did not induce any obvious alteration in cyclin D1 expression after 8 hrs (Fig. 2A). The average expression levels of cyclin D1 in injected cells at 8 hrs (not shown) and 24 hrs (Fig. 2B) confirmed that the there was no change in the cyclin D1 profiles in cells expressing an excess of inhibitory AKT protein.

It is possible that proliferative signaling molecules other than those inhibited above might be responsible for S phase cyclin D1 suppression. To test this possibility proliferative signaling in general was disrupted by serum withdrawal for 4 hrs. In this case, the level of Thr-286 phosphorylation of cyclin D1 was directly determined with a phosphorylation site-specific antibody (the generous gift of Michelle D. Garrett and David R. Mason), and compared to total cyclin D1. To avoid rapid degradation of phosphorylated cyclin D1 [27], the cells were treated with the proteasomal inhibitor MG132 for 2 hrs prior to analysis. Serum withdrawal for 4 hrs slightly reduced both total and Thr-286-phosphorylated cyclin D1. Significantly, however, the ratio of phospho/total cyclin D1 was not changed by serum deprivation in any cell cycle phase (Fig. 1B). This indicates that the overall rate of cyclin D1 Thr-286 phosphorylation is not altered in any cell cycle phase by disruption of proliferative signaling following serum removal. Taken together, the above results fail to support the notion that alterations in proliferative signaling through the cell cycle are responsible for the cell cycle specific expression pattern of cyclin D1.

### The activity of critical signaling molecules remains constant through the cell cycle

**Figure 3:**
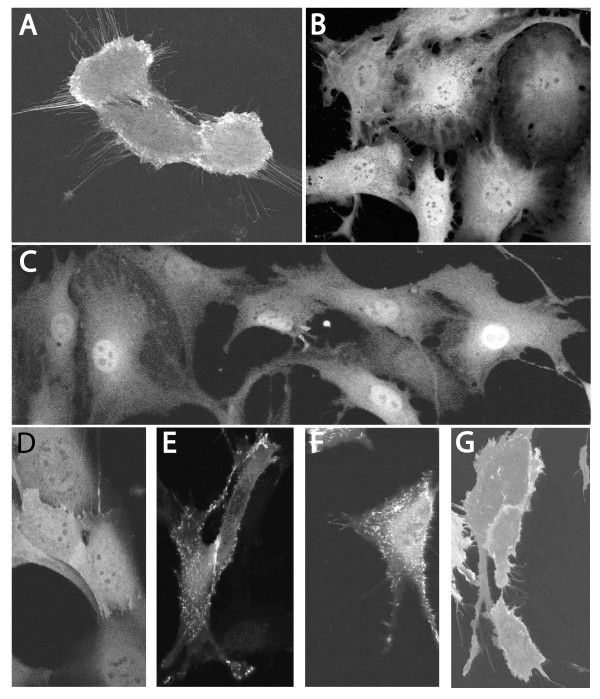
PI3K activity through the cell cycle. NIH3T3 cells were deprived of serum for approximately 30 hrs and injected with the PH-AKT-GFP plasmid. 15 hrs later cells were photographed under confocal microscopy either 20 min following serum stimulation (A), or without stimulation (B). Similarly, (C) cycling NIH3T3 cells were injected with the plasmid and photographed 15 hrs later. To confirm that the cycling cells retained the ability to stimulate PI3K activity, they were injected with the plasmid and deprived of serum for 0 hr (D), 5 hrs (E), 9 hrs (F) or 12 hrs (G) prior to addition of serum 20 min before confocal microscopy. Note the concentrations of fluorescence in cytoplasmic projections (E, F) or uniformly over the plasma membrane (G) following serum stimulation. The appearance of stimulated cells is clearly illustrated in a complete series of confocal sections at varying magnifications [presented in Additional files, movies 1, 2, 3].

To extend the above observations, we designed experiments to directly analyze the activity of individual proliferative signaling molecules through the cell cycle, to determine if there are alterations in proliferative signaling activity sufficient to account for the proposed variations in GSK3 activity. We first analyzed the activity of PI3K in individual cells of an asynchronous NIH3T3 culture using a plasmid expressing the PH domain of AKT linked to GFP [28]. T. Balla and associates have shown that the resulting protein strongly binds phosphatidylinositol-3-phosphate, the lipid product of PI3K activity, resulting in its association with the plasma membrane upon activation of PI3K [28]. To directly visualize the redistribution of fluorescence at the time of PI3K stimulation, the AKT-PH-GFP plasmid was microinjected into quiescent NIH3T3 cells. The intracellular distribution of fluorescence was determined by confocal analysis 15 hrs later without serum addition, or 20 min following addition of 10% serum to the culture. Without serum addition, the fluorescence was uniformly distributed throughout the cytoplasm and nucleus (Fig. 3B). Upon serum stimulation, however, the fluorescence became associated with the plasma membrane (Fig. 3A), including particularly high concentrations associated with tiny projections on the plasma membrane. Thus, the loss of fluorescence from the nucleus and cytoplasm, coupled with its redistribution to plasma membrane structures was diagnostic for PI3K activity [see Additional file, movies 1, 2, 3].

This assay was then applied to actively cycling NIH3T3 cells, where we found that the fluorescence of all cells remained evenly distributed throughout the cytoplasm and nucleus. Specific association of fluorescence with the plasma membrane was not observed in any of these cells (Fig. 3C). To demonstrate that cycling cells did retain the ability to produce a high level of PI3K activity, the PH-AKT-GFP plasmid was injected into proliferating NIH3T3 cells, after which serum was removed from the culture for 5–12 hrs, and then added back. Within 20 minutes of the addition of serum back to these cultures the fluorescence became associated with the plasma membrane, characteristic of PI3K activation described above for quiescent cells (Fig. 3D–G).

These results present an interesting model of proliferative signaling in cycling cells. The high levels of PI3K activity observed following serum addition to quiescent cultures most likely represents the response to a change in growth condition rather than a normal consequence of cell cycle progression. While PI3K activity is present in and required for the proliferation of actively cycling cells, the levels required are apparently much lower than observed upon serum stimulation. Importantly, there was no evidence of alterations in PI3K activity during S phase or any other cell cycle period in asynchronous cultures, reducing the likelihood that such an alteration might be responsible for the elevation in GSK3 activity, and the corresponding decline in cyclin D1 levels during S phase.

### AKT is activated uniformly through S and G2 phases

**Figure 4:**
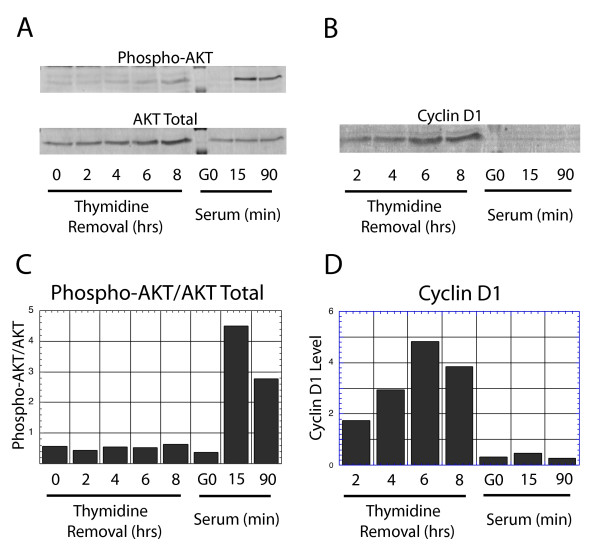
AKT activity in S and G2 phases: (A) NIH3T3 cells were synchronized with thymidine treatment in S phase and released by thymidine removal for the indicated times. In addition, quiescent cells were left unstimulated (G0), or stimulated with serum for the indicated times. In each case, lysates were collected and probed with an antibody specific for the activating phosphorylation on AKT. This was compared to western analysis of total AKT protein. Cells enter G2 phase 4–5 hrs following thymidine removal. (C) The levels were quantitated, and the ratio of phospho-specific AKT divided by total AKT levels is presented. (B) For comparison, the levels of cyclin D1 were analyzed by western analysis in the same lysates, (D) and quantitated.

The above study of individual cells in an actively cycling culture was next confirmed by biochemical analyses in synchronized cell populations. A single treatment of NIH3T3 cells with 2 mM thymidine has been shown to result in their synchronization in S phase. Following removal of thymidine, treated cells remain in S phase for 4 hrs, and enter G2 phase approximately 5 hrs later [24,27]. Critically, we have confirmed that the cell cycle expression profile of cyclin D1 in such synchronized cultures behaves as in asynchronous cultures, with low levels in S phase cells, followed by increasing levels upon entry into G2 phase (see Fig. 4D). Cultures prior to or at various times following release from thymidine blockage were tested by western analysis for the activating phosphorylation of AKT (Fig. 4A,C), while cyclin D1 expression levels are presented for comparison (Fig. 4B,D). No evidence of alterations in the activation level of AKT was apparent at any time following release from thymidine blockage, as the cells passed from S to G2 phase. As a control, the levels of the activating phosphorylation on AKT were seen to increase dramatically within 15 and 90 min following treatment of quiescent NIH3T3 cells with serum. Moreover, in the cultures which showed no difference in AKT activation levels, the levels of cyclin D1 protein were seen to increase during passage from S to G2 phase (as previously demonstrated [27]). We conclude that alteration in the activity of AKT is not responsible for suppression of cyclin D1 levels during S phase.

### GSK3 activity is constant in synchronized S and G2 phase cells

**Figure 5:**
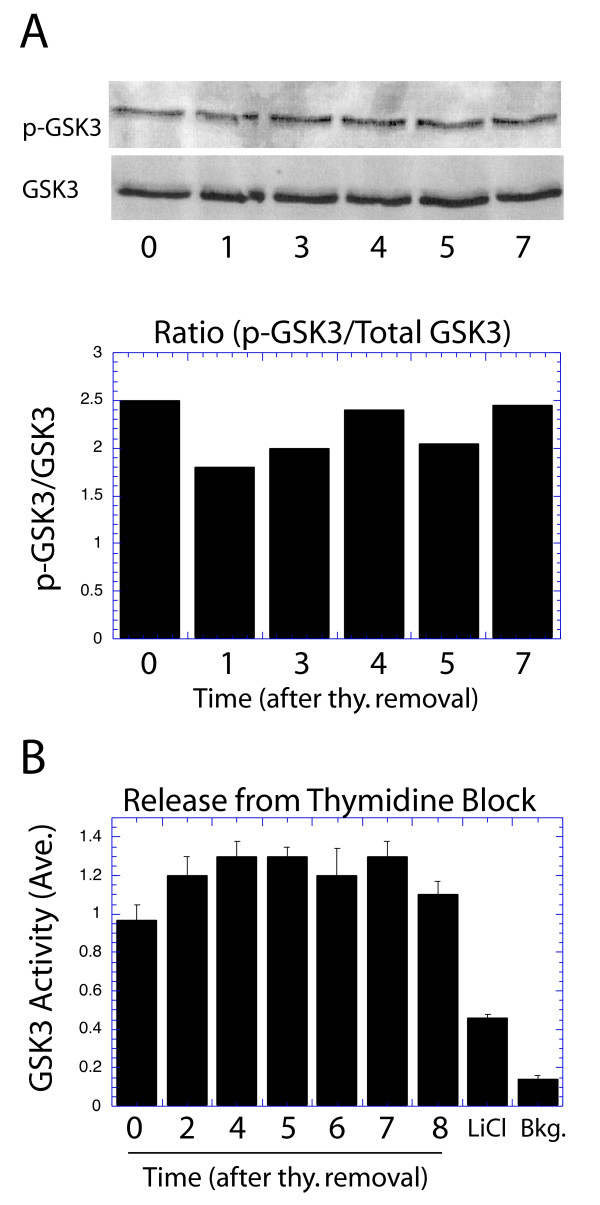
GSK3 activity does not vary between S and G2 phases: NIH3T3 cells were synchronized with thymidine treatment and released by thymidine removal for the indicated times. Cells remain in S phase for the first four hrs after thymidine removal and then enter G2 phase thereafter. (A) Cell lysates were collected at the indicated times following thymidine removal, and subjected to Western analysis for GSK3β phosphorylated on position 9, together with total GSK3 levels (upper panel). The results were quantitated, and the ratio of phosphorylate to non-phosphorylate GSK3 is presented (lower panel). (B) GSK3 protein was immunoprecipitated from cell lysates collected at the indicated times following thymidine release and the GSK3 protein assayed for kinase activity against a synthetic substrate. This figure represents the combined results of 10 different analyses, normalized to the level of GSK3 activity in thymidine-blocked cells. For comparison, S phase lysates were treated with 50 mM LiCl.

From the above results it is clear that the proliferative signaling pathway upstream of GSK3 does not vary through the cell cycle. However, it is possible that a pathway other than PI3K/AKT is responsible for regulation of GSK3 activity [29]. Therefore, antibody staining as well as biochemical analyses were performed to directly study variation of GSK3 activity through the cell cycle. NIH3T3 cells were synchronized in S phase by thymidine treatment and released as described above. Most of the cells progressed from S to G2 phase 5 hours after the release. The cells were collected at the indicated times, and the GSK3 phosphorylation was analyzed by western analysis with an antibody specific to the inhibitory phosphorylation of GSK3β on position 9 (Fig. 5A, upper panel). Total GSK3 was also determined and the phospho-GSK3/total GSK3 ratio presented (Fig. 5A, lower panel). We next directly determined the activity of GSK3 by synchronizing cells as described above, and immunoprecipitating GSK3 at the indicated times following release. The immunoprecipitated protein was then assayed with a synthetic peptide substrate whose phosphorylation was analyzed by PAGE analysis. Due to the low level of activity of GSK3 in NIH3T3 cells, this assay was repeated 10 times to obtain consistent results (Fig. 5B). There was some increase in GSK3 activity immediately upon release from thymidine blockage, but no evidence of any alteration upon passage from S to G2 phase. For comparison, there was a reduction in activity upon addition of serum to quiescent cultures, while the levels of GSK3 activity declined only gradually following serum removal from actively cycling cultures [see Additional file 4]. As a control, the inhibition of GSK3 activity by 50 mM LiCl is demonstrated (Fig. 5B). It is clear from these results than neither the upstream signaling pathway leading to GSK3 control, nor the activity of GSK3 itself varies during passage from S to G2 phase in synchronized cultures, or in actively cycling cells. This places doubt on the role of proliferative signaling or GSK3 activity in suppressing cyclin D1 levels during S phase.

### Inhibition of GSK3 activity has no effect upon cyclin D1 expression

**Figure 6:**
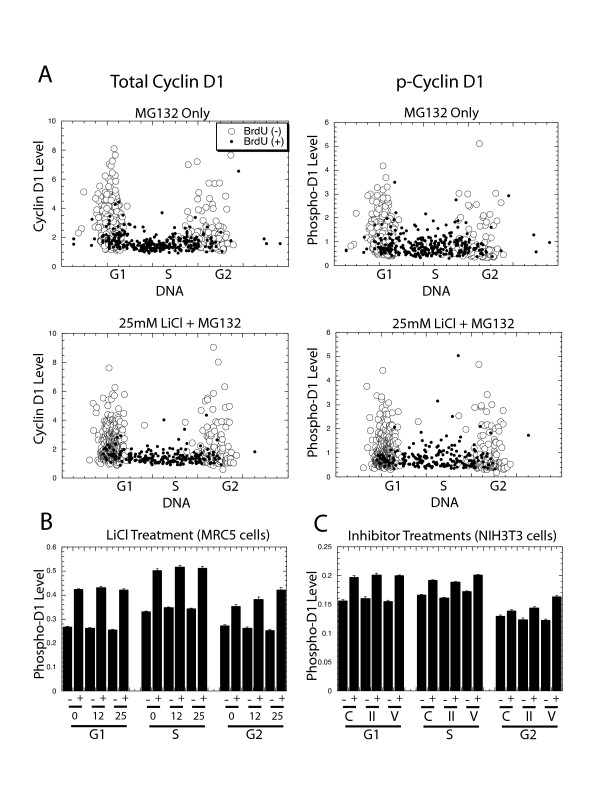
Inhibition of GSK3 does not alter cyclin D1 levels: (A) MRC5 cells were treated for 3 hrs with MG132 alone, or in combination with 25 mM LiCl. The cells were then fixed and the level of cyclin D1 (left), or the level of phospho-Thr-286 cyclin D1 (right), for each cell was determined by image analysis and plotted against its level of DNA (with BrdU positive cells indicated as small, closed circles). (B) In an analogous experiment, MRC5 cells were treated with the indicated concentration of LiCl together with MG132. After 3 hrs the average level of phospho-Thr-286 cyclin D1 in each cell cycle phase was determined for each LiCl concentration indicated (0, 12.5, of 25 mM). (C) NIH3T3 cells were treated with MG132 together with 25 mM valproate (V), with 25 μM GSK3 inhibitor II (II), or with no treatment as a control (C). The average levels of phospho-Thr-286 cyclin D1 in each cell cycle phase of each treatment is shown.

Experiments were next designed to determine if GSK3 activity plays a role in cyclin D1 expression or phosphorylation in any cell cycle phase. GSK3 activity was inhibited by 25 mM LiCl, and the proteasomal inhibitor MG132 was added to allow the accumulation of phosphorylated cyclin D1. To determine if the inhibition of GSK3 would reduce the phosphorylation of cyclin D1, the effect of this treatment upon phospho-Thr-286 accumulation was determined by image analysis following staining with the phospho-Thr-286-specific antibody [27]. LiCl influenced neither the total amount of cyclin D1 nor its level of phosphorylation following staining of human diploid fibroblast MRC5 cells. This is apparent from the cell cycle expression profiles (Fig. 6A), and from average levels determined from these profiles (Fig. 6B). To confirm the results with LiCl, we performed similar experiments on NIH3T3 cells with two other GSK3β inhibitors, sodium valproate [30] and GSK3 inhibitor II from Calbiochem. Neither of these treatments altered the ratio of total cyclin D1 to phosphorylated cyclin D1 (Fig. 6C).

**Figure 7:**
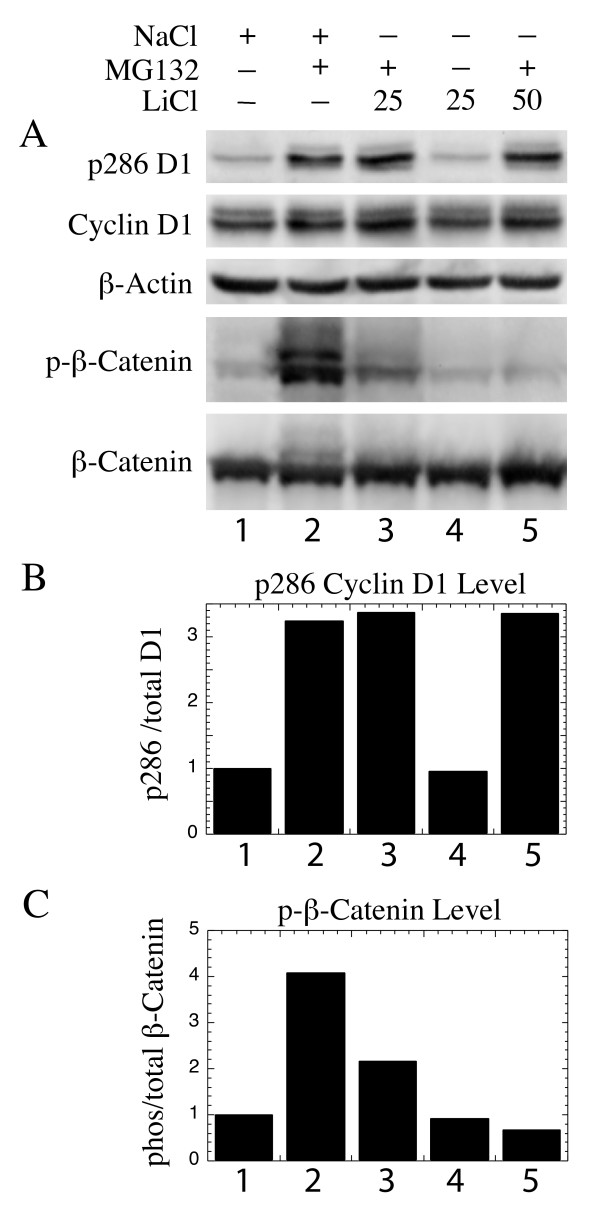
LiCl inhibits phosphorylation of β-catenin but not cyclin D1: NIH3T3 cells were cultured in medium containing 25 mM LiCl (columns 3, 4), 50 mM LiCl (column 5), or control medium with NaCl (columns 1, 2). In some cultures MG132 was added to block degradation of phosphorylated proteins (columns 2, 3, 5; see legend at the top). After 4 hrs lysates were prepared and probed for β-catenin, β-catenin phosphorylated on Ser 33/37/Thr 41, cyclin D1, cyclin D1 phosphorylated on Thr-286, and for actin as a loading control. (A) Photographs of the western results are presented. Each band was then quantitated, and (B) the ratio of phosphorylated cyclin D1 divided by the level of total cyclin D1 protein was determined and plotted immediately below the corresponding western bands. In addition, (C) the ratio of phosphorylated β-catenin divided by the level of total β-catenin protein is presented. Quantitative results correspond to the bands displayed in this figure immediately above them (with the same lane numbers).

This critical result was next confirmed by western analysis. NIH3T3 cultures were treated with medium containing 25 mM or 50 mM LiCl to inhibit GSK3 activity, or with medium containing equivalent amounts of NaCl as a control. MG132 was added to block degradation of phosphorylated proteins as above. After treatment for 4 hrs the levels of total and phosphorylated β-catenin were determined by western analysis with appropriate antibodies, while actin was analyzed as a loading control. β-catenin is a well characterized substrate of GSK3. Its phosphorylation would, therefore, serve as an indication of the activity of GSK3 within the treated cells. Since the lysate was not separated into nuclear and cytoplasmic fractions [31,32], little alteration in the total β-catenin levels were seen (Fig. 7A). On the other hand, high levels of phosphorylated β-catenin were observed in cells cultured in normal medium (Fig. 7 column 2). Phosphorylated β-catenin levels were dramatically reduced by 25 mM LiCl (Fig. 7 column 3) and almost completely eliminated by treatment with 50 mM LiCl (Fig. 7 column 5). Levels of the phosphorylated proteins were observed only when their degradation was blocked by treatment with the proteasome inhibitor, MG132 (Fig. 7 columns 3, 4). The results were confirmed by quantitating the ratio of phosphorylated to total β-catenin (Fig. 7C). On the other hand, in these same lysates neither concentration of LiCl had any effect upon the phosphorylation of cyclin D1 on Thr-286 (Fig. 7 column 3, 5). This conclusion was confirmed following quantitation of the western bands to determine the ratio of phosphorylated to total cyclin D1 levels (Fig. 7B). The fact that LiCl was able to block the phosphorylation of a well known GSK3 substrate, without influencing the phosphorylation of cyclin D1within the same cells, confirms the above conclusion that GSK3 is not a major kinase for cyclin D1 in these cells.

**Figure 8:**
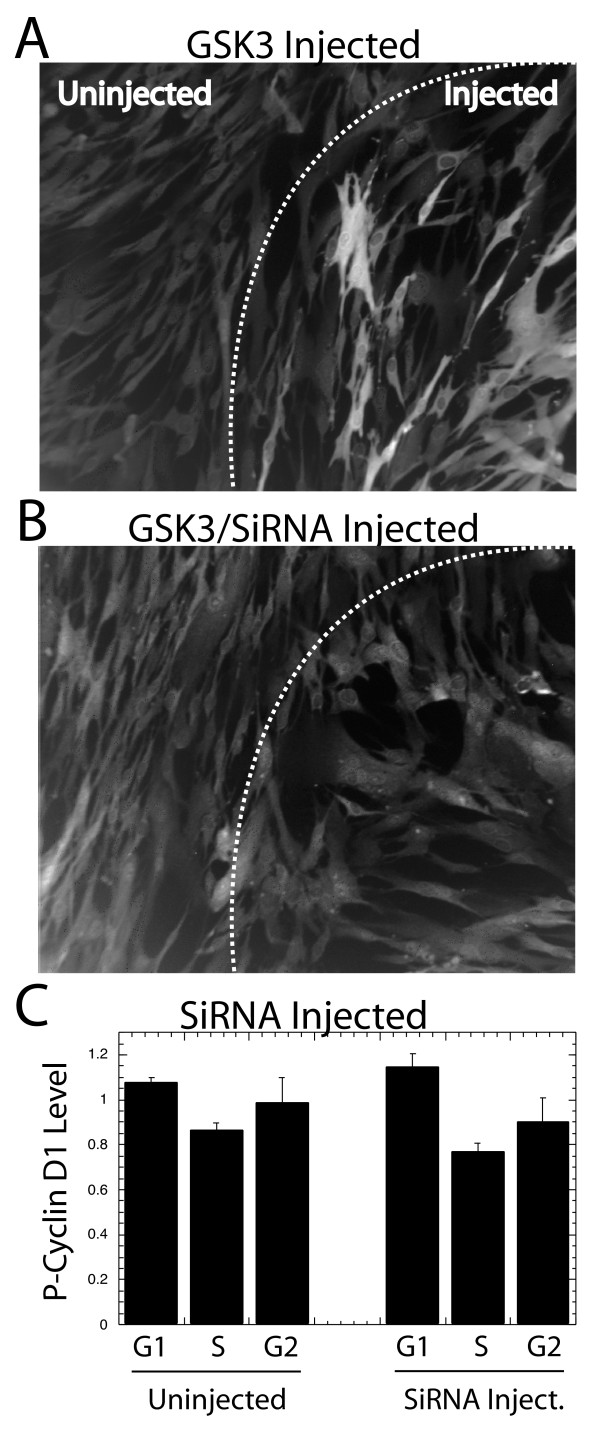
siRNA against GSK3 does not influence cyclin D1 phosphorylation: (A) A vector expressing activated GSK3β protein was injected into an NIH3T3 plate within a circular area marked on the back of the coverslip and indicated in the photographs above. (B) A similar injection of GSK3β was preceded 15 hrs earlier with an injection of an siRNA mixture against GSK3 α and GSK3 β. 5 hrs after these GSK3 plasmid injections cells were fixed and the GSK3 stained with a fluorescent antibody stain. The fluorescence photographs of each injection are presented. (C) To determine the influence of reduced GSK3 expression on the phosphorylation of cyclin D1, this siRNA was microinjected 15 hrs prior to a pulse with BrdU, fixation and staining for phospho-Thr-286 cyclin D1, BrdU and DNA. The average phospho-Thr-286 cyclin D1 levels in each cell cycle phase of injected, or neighboring uninjected cells is presented. MG132 was added 2 hrs prior to fixation to preserve phosphorylated cyclin D1 as above.

To strengthen this conclusion, GSK3 protein was ablated with a commercial siRNA mixture reported to suppress levels of both GSK3 α and GSK3β. To confirm its effectiveness, this siRNA was injected into NIH3T3 cells 12 hrs prior to a second injection of a plasmid expressing the GSK3 β gene (the gift of J. R. Woodgett). Following these injections the cells were fixed and stained with a fluorescent antibody stain against GSK3. While the plasmid expressed high levels of GSK3 when injected alone (Fig. 8A), prior injection with siRNA totally blocked expression of the high exogenous levels of GSK3 (Fig. 8B). Even though the endogenous levels of GSK3 are low, these endogenous levels were also suppressed by the injected siRNA (Fig. 8B). We conclude that the siRNA is able to efficiently ablate GSK3 expression. This siRNA was injected into NIH3T3 cells, which were left over night prior to treatment with MG132 and staining with the phospho-Thr-286 cyclin D1 antibody. The siRNA had no influence upon phosphorylation of cyclin D1 (Fig. 8C), or upon total cyclin D1 levels (not shown). We conclude that inhibition of GSK3 does not alter normal cell cycle regulated phosphorylation of cyclin D1.

### Over-expression of activated GSK3 β does not suppress cyclin D1 levels

**Figure 9:**
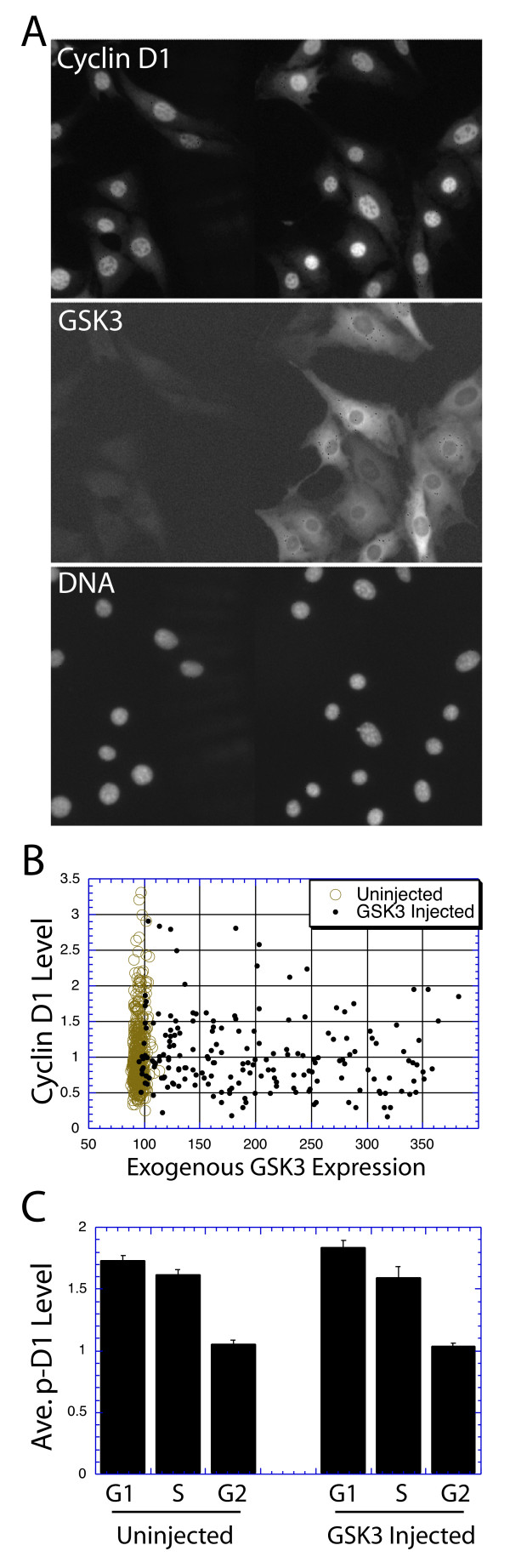
Activated GSK3 does not alter cyclin D1 levels: (A) A plasmid expressing an activated mutant of GSK3β was microinjected into the NIH3T3 cells at the right side of the area of cells photographed. 8 hrs following injection the cells were fixed and stained for GSK3, cyclin D1 and DNA. Separate fluorescence photographs of the same area of cells for of each fluorochrome are presented. (B) An injection of the GSK3 plasmid into NIH3T3 cells as above was performed and the expression of GSK3 determined by image analysis and plotted vs. the level of cyclin D1 in each cell. Injected cells are shown as small, solid circles. (C) An injection as described above was performed, but the cells were treated with MG132, and stained for phospho-Thr-286 cyclin D1 and BrdU. The average levels of phospho-cyclin D1 are plotted for each cell cycle phase for injected and neighboring uninjected cells.

The influence of GSK3 upon cyclin D1 degradation in NIH3T3 and MRC5 cells was next directly analyzed by microinjection of a plasmid expressing a constitutively active GSK3 β protein (S9A: the gift of J. R. Woodgett) [33,34]. Injected cells were stained for GSK3 and cyclin D1 at 8 and 24 hrs following injection. GSK3 staining was strong in injected cells at both time points, and displayed a cytoplasmic localization in most of the cells; whereas the staining was close to background level in the uninjected cells, indicating that the exogenous GSK3 level is far above the endogenous level (Figure 9A). However, the exogenous, constitutively active GSK3β did not apparently alter cyclin D1 levels at 8 hrs (Fig. 9A) or 24 hrs (not shown) following injection.

The effect of injected GSK3β upon cyclin D1 expression was next analyzed quantitatively. At 8 hrs following injection of the GSK3β expression plasmid cells were fixed and stained with fluorescent antibodies against cyclin D1 and GSK3. The intensity of each stain in each cell was determined by quantitative image analysis, and the GSK3 levels were plotted vs. cyclin D1 levels (Fig. 9B). The basal cyclin D1 level was apparent from the analysis of uninjected cells. This level of cyclin D1 expression was altered little if at all in cells containing even high levels of exogenous GSK3β. We conclude that GSK3β has little influence over cyclin D1 levels in NIH3T3 cells 8 hrs following injection. Similar results were obtained at 24 hrs in NIH3T3 cells, and in MRC5 cells (not shown). Finally, the average expression level of phospho-Thr-286 cyclin D1 was determined in each cell cycle phase following injection of the GSK3β plasmid as above, and compared to uninjected cells. In no case was there any difference between injected and uninjected cells in the level of phospho-Thr-286 cyclin D1 (Fig. 9C).

### GSK3 is potentially involved in G2 phase

**Figure 10:**
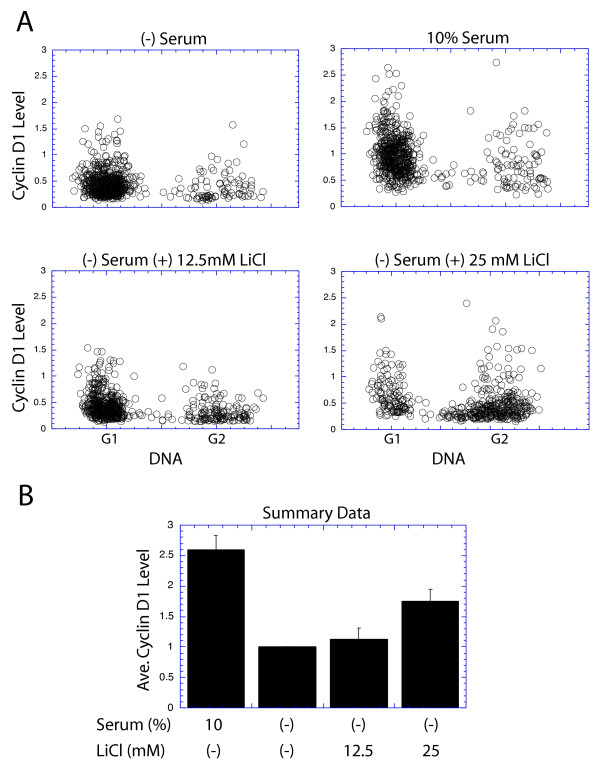
GSK3 regulation of cyclin D1 expression in the absence of serum: NIH3T3 cells were cultured in the indicated medium, following an initial pulse with BrdU. After 11 hrs the cells were fixed, stained and analyzed. (A) The cyclin D1 level of each cells is plotted vs. its DNA content, but only cells labeled with BrdU, and therefore in S phase at the beginning of the treatment period are displayed. (B) Three identical experiments were performed and the average level of cyclin D1 in G2 phase cells following all treatments is presented. For this analysis the value for serum-deprived cells was set at 1.0, and the relative expression levels following other treatments is displayed.

The above experiments show no evidence for the involvement of GSK3 in cyclin D1 expression or phosphorylation in any cell cycle phase. It should be emphasized, however, that all of those studies were performed upon cells cultured with normal growth factors which might have induced proliferative signaling within the cells to neutralize all GSK3 activity, thereby masking its potential role in regulating cyclin D1 in the absence of serum. This possibility is supported by the fact that while cyclin D1 suppression following serum removal is primarily due to reduced mRNA stability, there is also evidence for a limited role of altered protein stability upon serum removal [24]. To test the possibility that GSK3 might play a role in cyclin D1 regulation in the absence of serum, NIH3T3 cells were given a brief pulse of BrdU prior to serum removal for 11 hrs. Thus, cells in S phase at the time of serum removal would be labeled with BrdU. It is known that these cells would progress normally through mitosis, but with low cyclin D1 levels [22]. To determine if GSK3 might play a role in maintaining cyclin D1 at low levels in these serum-deprived cells, they were treated with 12.5 or 25 mM LiCl to inhibit GSK3 activity at the time of serum removal. Our attention was focused upon the BrdU labeled cells to determine if their cyclin D1 levels remained low upon entry into G2 phase. The addition of 12.5 mM LiCl failed to alter cyclin D1 levels, but 25 mM LiCl treatment resulted in the elevation of cyclin D1 levels in some of the cells passing from S to G2 phase in the absence of serum (Fig. 10A). Three duplicate experiments were performed and compared to demonstrate a significant increase in G2 phase cyclin D1 levels in serum deprived cells cultured with 25 mM LiCl (Fig. 10B). We interpret this result as potential evidence for a limited role of GSK3 in regulating G2 phase levels of cyclin D1, particularly when proliferative signaling is limited. This conclusion, however, is complicated by the fact that 25 mM LiCl slowed passage of cells into mitosis, resulting in an extended G2 phase (see Fig. 10A). We do not know if this extended G2 length might contribute to the slight elevation of cyclin D1 levels induced by LiCl in this experiment.

## Discussion

The fact that cyclin D1 levels are suppressed during S phase as a result of phosphorylation on Thr-286 [27] suggests that the kinase responsible might be GSK3 (see [15,17]). If GSK3 were responsible for the cell cycle specific suppression of cyclin D1 levels, the activity of this kinase and those signaling molecules which regulate its activity would be expected to vary in activity through the cell cycle. Thus, inhibition of these signaling molecules would be expected to abolish the cell cycle variations in cyclin D1 levels. We found, however, that inhibitors of PI3K, rapamycin and serum removal all suppressed cyclin D1 levels, but that this suppression was uniform through the cell cycle. The typical cell cycle expression pattern of cyclin D1 was not altered by any of these treatments. Moreover, cyclin D1 expression was not significantly suppressed in any cell cycle phase by expression of a dominant inhibitory AKT mutant protein, which is able to directly regulate GSK3 activity. These results fail to support the notion that these signaling molecules are responsible for specific suppression of cyclin D1 during S phase. To extend this result, the activity of signaling molecules was tested throughout the cell cycle. PI3K activity was assessed in individual cells with a GFP chimeric protein able to bind phosphatidylinositol-3 phosphate. The activating phosphorylation of AKT and the inactivating phosphorylation of GSK3β, together with the enzymatic activity of total GSK3, were assessed biochemically in synchronized populations. There was no evidence that any of these signaling activities vary thorough the normal cell cycle.

These results not only raise questions regarding the role of proliferative signaling in regulating cyclin D1 levels through the cell cycle, they bring into question the overall role of GSK3 in regulating cyclin D1 levels in living cells in general. To directly analyze the involvement of GSK3 in the control of cyclin D1 levels, its activity was inhibited by a variety of small molecule inhibitors, while its cellular levels were suppressed by siRNA. In no case was there any quantitative change in cyclin D1 levels in any cell cycle phase. LiCl failed to alter the phosphorylation of cyclin D1 on Thr-286 even though it efficiently blocked phosphorylation of β-catenin in the same cells. Finally, constitutively activated GSK3 β was introduced into living cells and found to have no influence upon cyclin D1 levels. We conclude that GSK3 does not play a role in the suppression of cyclin D1 during S phase, and that it is unlikely to be involved in the regulating cyclin D1 levels in the nucleus during any cell cycle phase. The one potential uncertainty with these results is the fact that in all the above experiments the cells were in the presence of normal serum levels. These might promote the constant suppression of GSK3 activity in these cells, masking the potential involvement of this enzyme in cyclin D1 regulation in the absence of proliferative signaling. In a final analysis, serum was removed from cycling NIH3T3 cells to allow GSK3 to become active, and then in some cultures it was again inhibited by the addition of LiCl. There was a slight, if minor elevation of cyclin D1 in a small proportion of G2 phase cells in the serum-deprived cultures treated with LiCl. It is not clear, however, if this was the direct result of cyclin D1 phosphorylation by GSK3, or the ability of GSK3 to interfere with passage through G2 phase.

The possibility that GSK3 might have an effect upon cell cycle progression is supported by its ability to influence a variety of cellular processes in addition to insulin and glycogen metabolism. It is directly involved in regulating cell proliferation and survival [33,34]. GSK3 is also able to phosphorylate transcription factors involved in growth regulation, including c-Myc, c-Jun and c-Myb [35]; and has been implicated in the action of growth factors on neural cell growth [36]. It is also able to regulate development directly [37], and as a member of the Wnt signaling pathway [38-41]. GSK3 is able to phosphorylate the Tau protein, involved in Alzheimer's disease [42]; play a role in mitotic spindle function [43]; repress Hedgehog signaling [44], and regulate cyclin E stability [45]. Recent studies implicate its action in cell polarity determination [46]. The results presented here suggest that the ability of GSK3 to phosphorylate a protein in vitro might not indicate that it has a role in the normal regulation of that protein within a living cell [47,48]. This conclusion, however, should be considered cautiously in cases where cell based assays indicate a connection between GSK3 and the regulation of a cellular protein [45].

Even though GSK3 has been implicated in a number of regulatory pathways, our view of GSK3 as a modulator of a wide variety of cellular processes must be reconsidered in light of recent genetic studies. With a reverse genetics approach in *Drosophila*, the inhibitory phosphorylation of GSK3 was shown to play a critical role in the action of the insulin/PI3K pathway, but was not involved in the stimulation of growth by PI3K [40]. In addition, knock-in studies in mice with homozygous activating mutations of both GSK3α and GSK3β demonstrated that while glycogen metabolism was altered in some tissues, the animals were otherwise normal in growth and development [41]. While developmental alterations can mask the normal role of a mutant protein, these studies emphasize the importance of carefully analyzing the role of GSK3 in signaling processes. In the studies reported here, we find that despite the fact that GSK3 is able to phosphorylate cyclin D1 on Thr-286 [15], this molecule does not play a role in the cell cycle regulated expression of cyclin D1. Our evidence further suggests that it may not play any major role in the expression of cyclin D1 in human and murine fibroblast cells. The possibility remains that GSK3 plays some role in other cell types, particularly during the growth factor induced increase in nuclear cyclin D1 during G2 phase. It has been shown, however, that ubiquitination of cyclin D1 can efficiently take place following phosphorylation of another site [49], or without the apparent requirement for phosphorylation [50,51]. Consistent with this conclusion, studies by others demonstrated that Thr-286 does not play a major role in the overall regulation of cyclin D1 levels in a variety of cells, although it is reported to play a role in the intracellular localization of this protein through the cell cycle [52]. Our studies on intracellular localization of cyclin D1 with the use of quantitative image analysis, however, have failed to produce any evidence of relocation from the nucleus to the cytoplasm during passage into S phase [20].

## Conclusion

These studies demonstrate that while the PI3K/AKT pathway plays a critical role in progression through the cell cycle, its activity in cycling cells is relatively low compared serum stimulated cells, and is invariant through the cell cycle. The cell cycle-specific effects of this signaling pathway reported previously [25,26], therefore, must result from the differences in the way target proteins are effected during different cell cycle phases. It is, thus, clear that the suppression of cyclin D1 during S phase is not the result of altered signaling during this cell cycle period. We conclude that increased phosphorylation of Thr-286 and reduced cyclin D1 stability are regulated by factors that are present throughout S phase, and in no other cell cycle period. This implies that the suppression of cyclin D1 levels during S phase will take place in every S phase regardless of the signaling environment of the cell, or the activity of any particular signaling molecule. This suppression not only allows the cell to actively synthesize DNA [23,27], but ensures that upon entry into G2 phase cyclin D1 levels are always low. Since the continuation of proliferation depends upon high levels of cyclin D1 during G2 phase, it would thus be necessary that the cell induce cyclin D1 levels each time it enters G2 phase if it is to continue proliferating. Thus, the apparently automatic suppression of cyclin D1 during each S phase ensures that signaling events of the previous cell cycle are erased, and forces the cell to re-evaluate the proliferative signaling environment prior to entry into the next cell cycle. These observations also have implications for tumor formation. Elevated cyclin D1 levels clearly play a central role in the stimulation of proliferation. It is possible that the simple over expression of cyclin D1, therefore, might lead to uncontrolled cell growth. The fact that its levels must decline during each S phase, however, limits the extent to which cyclin D1 levels can increase. Thus, since cyclin D1 has both positive and negative proliferative influences during different cell cycle phases, its regulation must remain relatively normal for cell growth to take place at all. This places severe limitations upon the extent to which a tumor cell can simply alter cyclin D1 levels as a means to achieve unrestrained growth properties [21].

## Methods

### Reagents

NIH3T3 and MRC5 cells used in the experiments were obtained from the American Type Culture Collection. Mouse monoclonal (72–13G) and rabbit polyclonal (H-295), anti-cyclin D1 antibodies were obtained from Santa Cruz Biotechnology, while a rabbit monoclonal anti-cyclin D1 was purchased from NeoMarkers Inc. Mouse anti-GSK3β and anti-PKBα/Akt antibodies were purchased from Transduction Laboratories. Phospho-GSK-3β (Ser9) antibody, GSK-3α/β siRNA was purchased from Cell Signaling Technology. Phospho-PKBα/Akt (Ser473) antibody was purchased from Promega. Anti-HA-fluorescein antibody was obtained from Roche. The rabbit polyclonal anti-phospho T-286 cyclin D1 antibody was raised as described previously [27], and was the generous gift of M. Garrett, or also purchased from Cell Signaling Inc. Mouse monoclonal anti-β-catenin was from BD Biosciences Pharmingen Inc., while rabbit polyclonal anti-phospho-β-catenin was from Cell Signaling Inc, and recognizes Ser 33/34 and Thr 41.

A plasmid expressing HA-tagged wild-type GSK3β was a kind gift of Dr. James R. Woodgett. A derivative plasmid expressing HA-tagged mutant GSK3β (S9A) was generated using QuikChange Multi Site-Directed Mutagenesis Kit (Stratagene). The plasmid pCDNA3-Akt-DN that expresses the dominant-negative form of AKT (K179 M) was kindly provided by Dr. Nissim Hay. The proteasomal inhibitor MG132, BrdU and sodium valproate were purchased from Sigma-Aldrich. LY294002, rapamycin, and GSK3 inhibitor II were obtained from CalBiochem. GSK3 Substrate (Phospho-Glycogen synthase peptide-2) and GSK3 non-substrate peptide (glycogen synthase peptide-2 (Ala21)) were purchased from Upstate Biotechnology.

### Cell culture

NIH3T3 mouse fibroblast cells were grown in DMEM medium supplied with 10% bovine calf serum. MRC5 human fibroblast cells were cultured in DMEM with 10% fetal calf serum. The cells used in all experiments were maintained at no more than 70% confluence. For S phase synchronization, NIH3T3 cells were cultured in growth medium containing 2 mM thymidine for approximately 14 h. To release the cells from the blockage, the monolayer was washed twice with PBS (37°C), then cultured with 10% bovine serum-containing DMEM for the indicated periods of time. Judging by the DNA content, BrdU labeling, and the incidence of mitotic figures, the culture was most enriched in G2 population around 5 h after the release. For G1 phase synchronization, NIH3T3 cells were serum-starved with 0.5% serum-containing DMEM for 48 h. The quiescent cells were stimulated to enter G1 phase by replacing the serum-deficient medium with 10% serum-containing medium. In the case were cells were treated with LiCl and MG132 together, the cells were pre-treated with LiCl for 15 minutes prior to addition of Mg132. Immunostaining procedures, kinase assay, and western blot analysis are described in the Additional files.

### Imaging

Digital images were collected with a cooled, monochrome camera (800 × 1000 pixels) from Roper Scientic with Metamorph software (Universal Imaging) at 200 magnication. The processing of images has been described [20]. Filter systems were designed by Chroma for the indicated fluorochrome. In summary, images of each fluorochrome were collected of the same area of the culture. Images of uniformly stained specimens were also photographed and used to shade the images collected, to correct for non-uniform illumination of the sample. After shading, the DAPI image was threshholded to identify the nuclei of each individual cell, and the resulting image used as a mask to measure the shaded images of each fluorochrome. Thus, the fluorescent intensity of each fluorochrome for each cell was identified. Results presented result from 100–2000 individual cells. Normally approximately 150–300 cells were injected for each experiment. S phase cells were identified as those labeled with BrdU, while G1 and G2 phase cells were BrdU negative and distinguished by DNA content. BrdU was added for 20–30 min as a pulse (5-Bromo-2'-deoxyuridine, Boehringer stock solution).

### Confocal and time-lapse analysis

Confocal analysis of GFP expression was performed with a Leica instrument on living cultures. The injected cells were identified by a circular mark on the back of the coverslip. Cells were localized under normal illumination so that illumination for confocal analysis would require a minimal exposure to laser light. The cells were mounted under a coverslip in the indicate medium and the procedure normally completed within 10 minutes. For fluorescence time-lapse, images were taken for 200 milliseconds every 1–2 hrs. This exposure did not interfere with the normal proliferation of the cells.

### Additional methods for additional files

#### Immunofluorensce staining of cyclin D1 and BrdU in monolayer cells

NIH3T3 at 50% confluence on coverslips were fixed with 100% methanol at room temperature for 15 minutes. The coverslips were washed with PBS two times and blocked with blocking buffer (1% of BSA in PBS) for 4 hours. For staining and image quantitation of cyclin D1, the method was that detailed previously [20]. Briefly, the blocked coverslips were incubated with cyclin D1 antibody (1:50 dilution) at 4C overnight, followed by three washes of PBS. The secondary antibody coupled with fluorochome (1:1000 dilution) was applied to coverslips at 37C for 3 hours at 4C overnight. Coverslips were then washed 3 times with PBS. BrdU staining was performed as previously described [53]. The coverslips were re-fixed with 100% methanol followed by two washes with PBS-0.5% Tween-20 and two washes of distilled water. Treatment of 1.5 NHCl was then applied to coverslips at room temperature for 10 minutes followed by two washes of PBS-0.5% Tween-20 at pH 7. The coverslips were re-blocked with CAS block solution (ZYMED) at room temperature for 2–4 hours, followed by incubation with BrdU antibody (1:300 dilution) at 4C overnight. After 3 PBS-Tween-20 washes coverslips were incubated with secondary antibody coupled with Cy5 in 0.3% BSA in PBS at 4C overnight. Further three washes were required before performing DAPI staining for DNA content.

### Kinase activity assays of GSK3

Kinase activity assay of GSK3 was done according to X. Fang et al. [29]. 60–70% confluent NIH 3T3 cells treated as indicated in the text were washed with cold PBS, scraped and collected in Eppendorf tubes. The cell pellets were snap frozen in liquid N2 and stored in -80C until all time points were collected. Pellets were lysed on ice for 30min in a lysis buffer containing 1% Triton-X-100, 50 mM Hepes, 150 mM NaCl, 1.5 mM MgCl_2_, 1 mM EGTA, 10% glycerol, 100 mM NaF, 10 mM sodium pyrophosphate, 25 mM beta glycerol phosphate, 1 mM DTT, 1 mM sodium vanadate, 1 mM benzamidine, 0.1 M okadaic acid, 10 g/ml aprotinin, 10 g/ml leupeptin and protease inhibitor cocktail (Sigma). The lysates were centrifuged at 14,000 at 4C for 30 min. The supernatants were aliquoted and stored at -20C.

The in vitro kinase assay of GSK3 was conducted in 40 μl of reaction mixture, which consisted of 75 μg of total protein, kinase reaction buffer (consisting of 10 mM 4-morpholinepropanesulfonic acid pH 7.4, 1 mM EDTA, 10 mM Mg Acetate, 50 mM beta glycerol phosphate, 20 mM MgCl_2_, 250 M cold ATP, 1 mM sodium vanadate, 0.5 mM NaF, 0.1 M okadaic acid, 1 mM benzamidine, 1 mM DTT and protease cocktail inhibitor), 62.5 mM phosphoglycogen synthase peptide-2 (Upstate Biotechnology) or glycogen synthase peptide-2 (Ala-21) (negative control for the substrate). The reaction was started by the addition of 2.5 μCi of [γ-^32^P] ATP. The mixture was incubated at 30C for 30 min, and briefly centrifuged. 15 μl of supernatant was spotted on Whatman P81 phosphocellulose paper. The filters were washed gently in 0.75 % phosphoric acid three times (5 min. each wash), and rinsed in acetone, dried and counted in a liquid scintillation counter.

### Phosphorylation assay of AKT/PKB

Cells were washed once with cold PBS and scraped, collected in Eppendorf tubes and briefly centrifuged. PBS as a supernatant was aspirated, the pellets were snap frozen in liquid nitrogen, and stored at -80C until all the time points were collected. The cells were lysed in lysis buffer (0.5% NP40, 50 mM HEPES (pH 7.6), 200 mM NaCl, 1 mM EDTA, 20 mM NaF, 10 mM -glycerophosphate, 0.1 mM sodium orthovanadate, 10 g/ml benzamidine, 1 mM PMSF and protease inhibitor cocktail (Sigma)) on ice for 30 min, followed by centrifugation in Eppendorf centrifuge (4C) at 14,000 rpm for 30 min. Supernatants were aliquoted and stored at -20C. 30 μg of total protein was run on a 10% SDS-PAGE gel and transferred onto a supported nitrocellulose membrane (Schleicher and Schuell) for 30 min using a semidry transfer apparatus (BioRad). The membrane was blocked for 1 hr at room temperature, using either 5% non fat dry milk or 1% BSA in Tris buffer saline containing 0.05 % tween-20 (TBST). It was then incubated in the primary antibody in overnight at 4C. The following dilutions were used for the primary antibodies: anti-phospho AKT ps473 (1:500); anti-phospho GSK3 (1:500); anti-AKT (1:500); anti-GSK3 (1:2000). The membrane was washed three times in TBST, 5 min per wash and then incubated in AP-conjugated anti-mouse IgG (1:1000) or anti-rabbit IgG (1:30,000) for 2 hours at room temperature. Following three washes with TBST, the blot was incubated with AP based fluorescent substrate (Amersham Pharmacia Biotech) and the bands were visualized and quantified by a fluorescence scanning instrument such as Molecular Dynamics Stormimager.

## Authors' contributions

KY performed the majority of the biochemical and cell analytical experiments, organized and analyzed data, and drafted the initial manuscript. YG designed and constructed plasmids and prepared DNA. WCS performed experiments utilizing confocal microscopy to localize phosphophatidyl-3-inositol. JH determined GSK3 and AKT activities biochemically through the cell cycle. JF performed photography and aided in image analysis. MH helped KY and DWS in design of experiments and data analysis, and performed some microinjections. DWS performed microinjections, finalized the manuscript and discussed it with the other authors.

## Supplementary Material

Additional file 1 The appearance of a stimulated compared to an unstimulated cell, low power. Two NIH3T3 cultures were serum deprived for 30 hrs, microinjected with the PH-AKT-GFP plasmid, and cultured for 14 hrs prior to stimulation of only one of the cultures with 10% serum 10 min prior to photography. This movie is a sequence of fluorescent confocal images beginning near the coverslip and extending through one cell from the stimulated culture, and one cell from the unstimulated culture. The distribution of the fluorescence at the plasma membrane and away from the interior of the cell is apparent in the stimulated cell (40× objective, 10 micron steps). Click here for file

Additional file 2 The appearance of a stimulated compared to an unstimulated cell, high power. An NIH3T3 culture was serum deprived, injected with the PH-AKT-GFP plasmid, and stimulated as above. Fluorescent confocal images (63X/1.4 objective) beginning at the coverslip and extending through the cell were taken of one cell 10 min following serum stimulation, and of another cell in an unstimulated culture prepared in parallel. The membrane structures observable following stimulation can be compared to the cytoplasmic localization in the unstimulated cell. Click here for file

Additional file 3 GFP localization through a single, stimulated cell. An NIH3T3 culture was serum deprived, injected with the PH-AKT-GFP plasmid, and stimulated as above. This series of fluorescent confocal images (40X/1.25 objective) illustrates the appearance of cytoplasmic structures that are at times visible in these stimulated cultures. Click here for file

Additional file 4 GSK3 activity in serum-deprived cultures. (A) NIH3T3 cells were synchronized by thymidine treatment and released for the indicated times prior to lysis and assay of the GSK3 activity. For comparison, NIH3T3 cells which had been deprived of serum for 48 hrs were analyzed for GSK3 activity without serum stimulation (0 hrs), and following serum stimulation for the indicated number of minutes. These are typical results of a single experiment. (B) To determine the effect of serum removal upon GSK3 activity, actively proliferating NIH3T3 cultures were deprived of serum for the indicated times prior to lysis and assay of GSK3 activity. Click here for file
