# Supplementary figures and images for: Glycogen synthase kinase 3 has a limited role in cell cycle regulation of cyclin D1 levels
Source: BMC Cell Biol. 2006 Aug 30;7:33. doi: 10.1186/1471-2121-7-33 (PMC1592484; doi:10.1186/1471-2121-7-33)

A

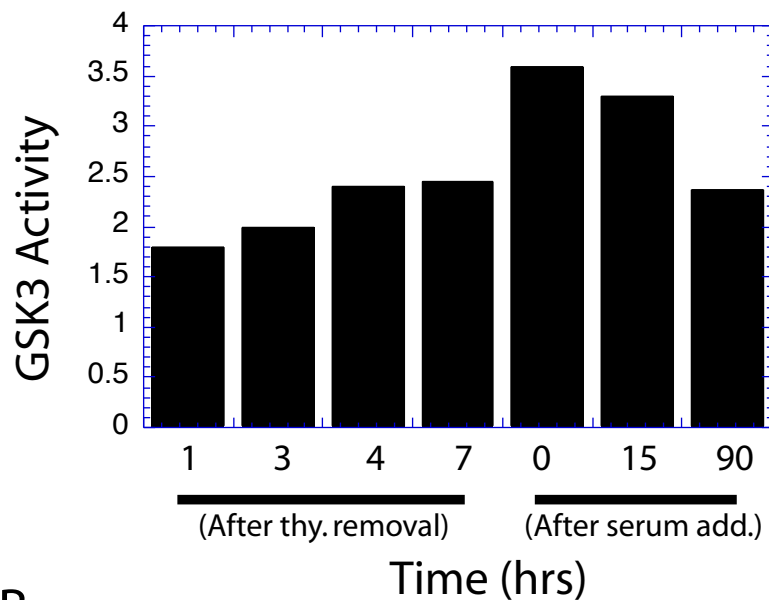

B

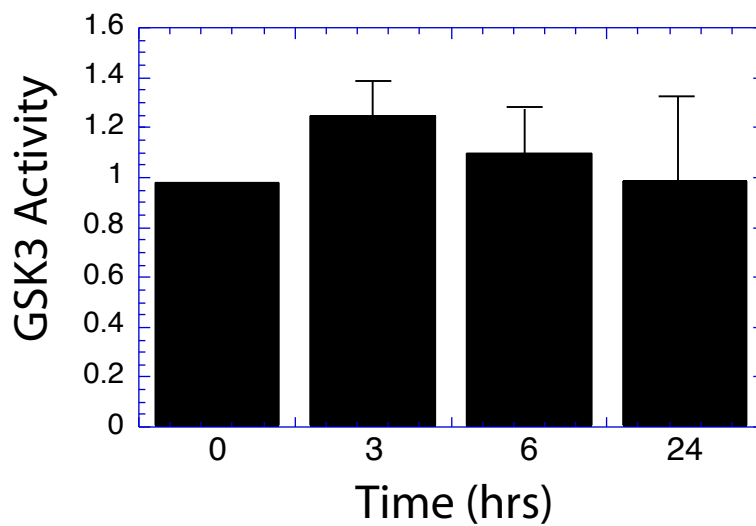

Supplementary Figure D

Supplement: Additional file 4 — GSK3 activity in serum-deprived cultures. (A) NIH3T3 cells were synchronized by thymidine treatment and released for the indicated times prior to lysis and assay of the GSK3 activity. For comparison, NIH3T3 cells which had been deprived of serum for 48 hrs were analyzed for GSK3 activity without serum stimulation (0 hrs), and following serum stimulation for the indicated number of minutes. These are typical results of a single experiment. (B) To determine the effect of serum removal upon GSK3 activity, actively proliferating NIH3T3 cultures were deprived of serum for the indicated times prior to lysis and assay of GSK3 activity. [file 1471-2121-7-33-S4.pdf]
